# Improvement of osseointegration efficacy of titanium implant through plasma surface treatment

**DOI:** 10.1007/s13534-022-00245-9

**Published:** 2022-08-30

**Authors:** Hyungyu Lee, Hyun Jeong Jeon, Ara Jung, Jinwoo Kim, Jun Young Kim, Seung Hun Lee, Hosu Kim, Moon Seop Yeom, Wonho Choe, Bomi Gweon, Youbong Lim

**Affiliations:** 1 Department of Nuclear and Quantum Engineering, Korea Advanced Institute of Science and Technology (KAIST), 34141 Daejeon, Republic of Korea; 2 Plasmapp Co., Ltd, 372 Dongbu-daero, 18151 Osan-si, Gyeonggi-do Republic of Korea; 3 Department of Mechanical Engineering, Sejong University, 05006 Seoul, Republic of Korea; 4 Seoul Top Dental Clinic, 345 Omok-ro, Yangchun-gu, 07999 Seoul, Republic of Korea

**Keywords:** Dielectric barrier discharge, Hydrocarbon, Titanium implant, Osseointegration, Surface treatment

## Abstract

A novel plasma treatment source for generating cylindrical plasma on the surface of titanium dental implants is developed herein. Using the titanium implant as an electrode and the packaging wall as a dielectric barrier, a dielectric barrier discharge (DBD) plasma was generated, allowing the implant to remain sterile. Numerical and experimental investigations were conducted to determine the optimal discharge conditions for eliminating hydrocarbon impurities, which are known to degrade the bioactivity of the implant. XPS measurement confirmed that plasma treatment reduced the amount of carbon impurities on the implant surface by approximately 60%. Additionally, in vitro experiments demonstrated that the surface treatment significantly improved cell adhesion, proliferation, and differentiation. Collectively, we proposed a plasma treatment source for dental implants that successfully removes carbon impurities and facilitate the osseointegration of SLA implants.

## Introduction

Dental implants are generally used as artificial tooth roots to support prosthetic suprastructures ranging from single-crown to fixed and removable prostheses. Long-term and stable fixation are the most essential requirements for successful implantation; therefore, osseointegration is considered a critical implant fixation process [1]. The most commonly used implant material is titanium or titanium alloy, which is a gold standard for oral implant applications owing to its superior biocompatibility and ability to achieve osseointegration [2, 3]. Given that dental implants are in close and direct contact with bone tissues, the surface properties of titanium dental implants are the most important factors for effective osseointegration and long-term clinical success [4]. Rough surfaces are typically believed to be more crucial for osseointegration than smooth surfaces. Consequently, the topography and roughness of implant surfaces adjacent to bone tissues have continuously been improved to increase the long-term success rate [4–11]. Various surface treatment technologies, such as grit-blasting, acid-etching by mineral acids, and electrochemical anodic oxidation, have been developed to modify the surface roughness [12–14]. Recently, implants sandblasted with large grit followed by acid-etching (SLA) have become the global standard for enhancing surface roughness and facilitating osseointegration [6]. SLA implants have good clinical performance immediately after production, but surface aging owing to carbon contamination is inevitable because the implants are sterilized and exposed to the ambient air before being utilized in clinical practice. Furthermore, these carbon impurities have been shown to gradually accumulate on the implant surfaces, declining the surface bioactivity over time [15, 16].

The osseointegration process consists of a series of complex physiological processes, including extracellular matrix (ECM) protein adsorption, cell adhesion, migration, proliferation, and differentiation [17]. Therefore, the bioactivity of the implant surface can be confirmed by the protein adsorption and cell adhesion properties of the surface. In previous studies, it was proved that protein adsorption significantly decreased in implants with surface aging over time [18]. Notably, impurities like hydrocarbon contaminants can cause adverse effects on cellular adhesion, resulting in early marginal bone loss. Such impurities are often found in many commercially available implants, thus significant efforts have been made to resolve this problem.

In this context, ultraviolet (UV) light irradiation technology has been developed to reactivate aged or contaminated implant surfaces. The UV irradiation method for reactivating the surface of titanium dioxide (TiO_2_) was first suggested in 1997 and has been widely used to produce hydrophilic surfaces in environmental and clean-energy science [19]. Photoinduced hydrophilicity is known to be induced by the desorption of water molecules through surface-heating and partial removal of hydrocarbons through photocatalytic decomposition [20]. Under the same principle, UV irradiation technology can reduce the hydrocarbon from the implant surfaces and significantly increases protein adsorption and cell adhesion on the implant surface [21, 22]. However, since expensive crystalline packaging materials such as quartz must be used so that UV light can reach the implant surface, the economic efficiency of the implant is underminded. Moreover, the treatment time is at least 3 h, which drastically deteriorates the clinical usability.

To address these limitations, various plasma-based technologies have been recently developed, and plasma treatment has been proved to greatly reduce hydrocarbon impurities and increase osseointegration efficacy [23–30]. A chamber-type plasma treatment was proposed by Diener Electronic GmbH. For the chamber-type plasma treatment, the implant should be mounted on a specially designed implant holder to be processed inside a vacuum chamber. Additionally, this treatment method requires a long treatment time of at least 10 min while applying argon gas [24]. Jet-type plasma treatment methods proposed by other groups require an extra argon or nitrogen gas supply to generate jet plasma at atmospheric pressure [26, 29]. Moreover, due to the small plasma volume of the jet plasma, usually the these plasma treatments require continuous movement of the implant to uniformly treat the entire implant surface. Both currently available chamber-type and jet-type methods require an additional gas supply for plasma discharge; however, the additional gas supply requires installation of gas cylinders, gas piping systems, and continuous gas charging, which require considerable maintenance efforts. Therefore, it is necessary to improve these existing plasma sources to ensure more reliable and convenient clinical applications. In this study, we propose a novel plasma surface activation source that can discharge plasma inside the implant package to reduce contamination on the implant surface and improve osseointegration performance.

## Materials and methods

### Plasma setup

**Fig. 1:**
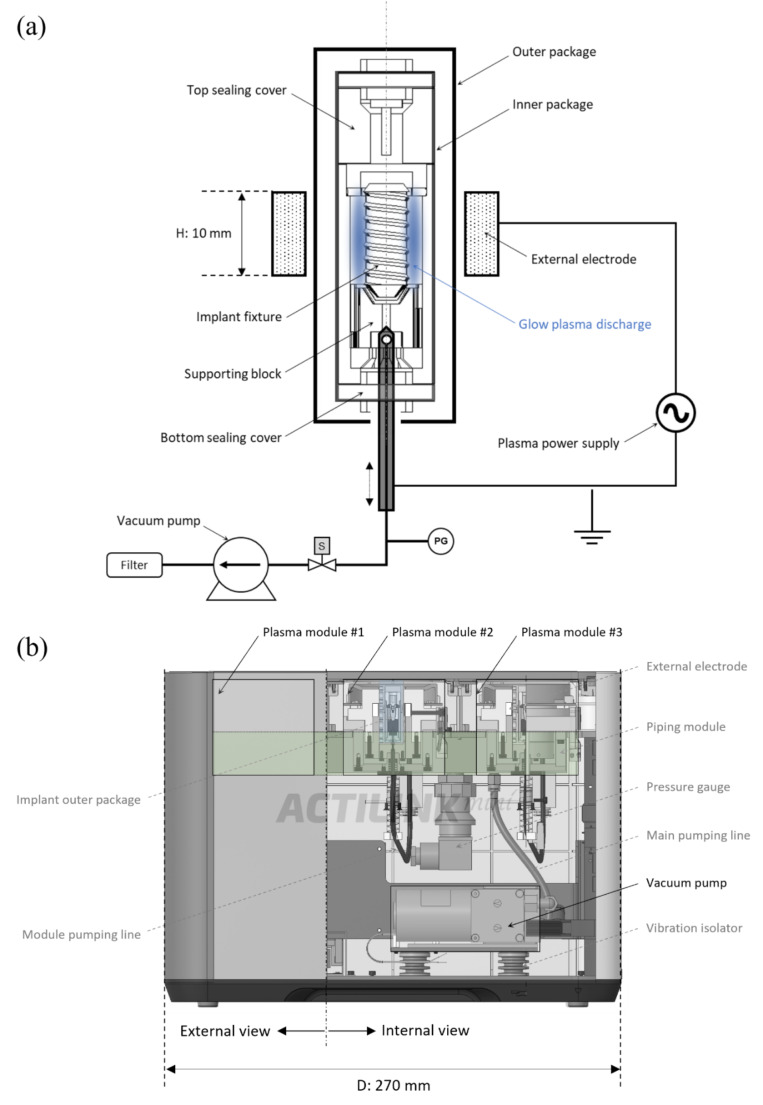
**a** Configuration of plasma source including implant and packaging, **b** plasma treatment system consisting of three identical plasma modules

The implant fixture and supporting block were electrically grounded, as shown in Fig. 1a. A sinusoidal electric power with a frequency of 100 kHz and voltage of 3 kV was applied to the external electrode to generate a dielectric barrier discharge (DBD) on the implant surface inside the inner package. The package wall functioned as a dielectric barrier layer made of transparent polypropylene. As depicted in Fig. 1a, a hollow needle was designed to reach the inner package through a bottom sealing cover made of silicone rubber, and the other end of this needle was connected to a diaphragm vacuum pump (N 84.3 ANDC, KNF) and served as a port to pump out gas. The pressure inside the package was stably maintained in the range of 5 to 10 Torr thanks to the elastic characteristics of the silicon rubber of the bottom sealing cover. As shown in Fig. 1b, our plasma system (ACTILINK, Plasmapp) was equipped with three identical plasma modules, each of which was described in Fig. 1a. Plasma treatment could be processed independently in each module, with a shared vacuum pump and pressure gauge for the plasma modules to achieve a compact design with a width, depth, and height of 210, 370, and 270 mm, respectively. In this study, a commercial implant (SLA, Straumann) with dimensions of 4.1 mm in diameter and 10 mm in length was used, and the implant was repackaged to be processed by a plasma system with a total treatment time of 60 s.

### Numerical investigations

Numerical investigations were performed to find the optimal plasma discharges conditions to effectively remove hydrocarbon contaminants from the implant surface. COMSOL Multiphysics^®^ software was used to acquire the spatiotemporal behavior of DBD plasma using a symmetric two-dimensional geometry as the computational model. The geometry is identical to the actual plasma source described in Fig. 1a. The computational domain’s height is set to 2 mm, and the electron properties are found to be constant along the vertical direction. The electron density and temperature along the radial direction, which are critical parameters in hydrocarbon decomposition reactions, were calculated in numerical probes. The following equations computed the spatiotemporal dynamics of electron density:

1 $$\frac{\partial }{\partial t}\left({n}_{\tt e}\right)+ \nabla \bullet {\varGamma }_{\tt e}={R}_{\tt e}$$

2 $${\varGamma }_{\tt e}=-\left({\mu }_{\tt e}\bullet \tt \bf \overrightarrow{E}\right){n}_{\tt e}-\nabla \left({{D}_{\tt e}}{n}_{\tt e}\right)$$

where* n*_e_ is the electron density, *Γ*_e_ is the electron flux, *R*_e_ is the reaction rate of electrons, ${\mu }_{e}$ is the electron mobility, $\bf \overrightarrow{E}$ is the electric field, and ${D}_{e}$ is the electron diffusivity. The energy density of the electron was given by

3 $$\frac{\partial }{\partial t}\left({n}_{\tt \epsilon }\right)+\nabla \bullet {\varGamma }_{\tt \epsilon }+ \overrightarrow{\bf E}\bullet {\varGamma }_{\tt e}=0$$

4 $${\varGamma }_{\tt \epsilon }=-\left({\mu }_{\tt \epsilon }\bullet \overrightarrow{\bf E}\right){n}_{\tt \epsilon }-\nabla \left({{D}_{\tt \epsilon }}{n}_{\tt \epsilon }\right)$$

where ε is the electron energy density, *Γ*_ε_ is the electron energy flux, ${\mu }_{\tt \epsilon}$ is the electron energy mobility, and *D*_ε_ is the electron energy diffusivity. This model considered electrons, nitrogen molecules, and ions. The following equations, which were connected to the electron density and temperature, were used to calculate the ion density:

5 $$\frac{\partial {n}_{\tt i}}{\partial t}+\nabla \bullet {\varGamma }_{\tt i}={R}_{\tt i}$$

6 $${\varGamma }_{\tt i}=\left({\mu }_{\tt i}\bullet \overrightarrow{\bf E}\right){n}_{\tt i}-{ D_{\tt i}}\nabla {n}_{\tt i}$$

where *n*_i_ is the ion density, *Γ*_e_ is the ion flux, *R*_i_ is the reaction rate of the ion, ${\mu }_{\tt i}$ is the ion mobility, and ${D}_{\tt i}$ is the ion diffusivity. Poisson’s equation was used to calculate the electric field depending on the density of the electrons and ions:

7 $$\overrightarrow{\bf E}=-\nabla \varphi$$

8 $$\nabla \bullet \overrightarrow{\bf D}={\rho }_{\tt q}$$

where $\overrightarrow{\bf D}$ is the electric displacement field, φ is the electric potential, and ${\rho }_{\tt q}$ is the space charge density.

In this model, the same electric power conditions as those in the experimental setup were applied, and the implant was likewise electrically grounded. The initial concentration of the electron was set to 10^10^ m^−3^, and the initial conditions for the neutral molecules were probed parametrically in the range of 2–100 Torr.

### Hydrocarbon contamination analysis

The degree of hydrocarbon contamination was determined by X-ray photoelectron spectroscopy (XPS, Axix-Supra, Kratos). The ratio of carbon element to the surface of the implant without plasma treatment (SLA) and the implant with plasma treatment (SLA + Plasma) was analyzed and compared.

### Protein adsorption assay

Following plasma treatment, both the SLA and SLA + Plasma groups were immersed in a trea-treated 96-well plate containing a fibronectin protein solution (50 µg/ml, #356,008, Corning). After 2 h of incubation at 37 °C, the implants were washed with phosphate-buffered saline (PBS) to eliminate any residual proteins that had not been adsorbed onto the implant surface. The adsorbed proteins were then lysed from the implant surfaces using 2% sodium dodecyl sulfate solution at 37 °C for 18 h. A Micro BCA™ Protein Assay Kit (#23235, Thermo Fisher Scientific) was then used to determine the protein concentration.

### Cell proliferation assay

The human osteosarcoma cell line, Saos-2, which was known to have osteoblastic features, was used in our study. Saos-2 was purchased from the Korean Cell Line Bank (KCBL#80023). Saos-2 cells were cultured with Minimum Essential Medium (#LM007-01, Welgen) supplemented with 10% fetal bovine serum (#S001-01, Welgen) and 1% Anti-Anti (#15240-062, Gibco).

In a non-treated 96-well plate (#32096, SPL), a high concentration (10^6^*cells/well*) of Saos-2 cell suspension was prepared to mimic an environment comparable to bone tissue with high bone cell density. After plasma treatment, the SLA and SLA + Plasma were immersed in the Saos-2 cell suspension and incubated at 37 °C in a CO_2_ incubator for 2 h, for the cells to attach to the implant surface. After removing the SLA and SLA + Plasma samples from the cell suspension, they were gently washed three times using PBS buffer and immersed in a new medium to allow the cells to proliferate. The number of cells adhering to the implant surface was assessed at four different time points after attaching the cells on the SLA and SLA + Plasma: 2 h (0.1 day), and 1, 3, and 5 days. The 2 h time point data confirmed the initial cell adhesion efficiency, and the sequentially measured 1, 3, and 5 days time point data confirmed the proliferation efficiency. At each time point, the samples were gently washed using PBS to eliminate weakly adhered cells on the surface, placed in a culture medium containing Cell Counting kit-8 (CCK-8, #CK04, Dojindo), and left for 90 min to allow the CCK-8 reagent to respond.

### Cell differentiation assay

An alkaline phosphatase (ALP) assay kit was purchased from Abcam (#ab83369, Abcam). After seven days of cell culture, cells were washed with PBS and lysed twice with ALP assay buffer on ice for 30 min. The lysate was collected and centrifuged at 13,000 rpm and 4 °C for 15 min. Then, 80 µl of lysate was taken and placed into a 96-well plate, and 50 µl of 5 mM p-Nitrophenyl Phosphate (pNPP) solution was added. The lysate-pNPP mixture was incubated at 25 °C for 60 min in the dark. The reaction was stopped by adding a stop solution. The optical density (OD) was measured using a microplate reader at 405 nm wavelength. ALP activity was calculated by the pNPP standard curve.

### Image analysis of the implant surface

The implant surface of the SLA and SLA + Plasma was imaged by the scanning electron microscope (SEM, PHENOM XL). In order to precisely compare the surface before and after plasma treatment, we attempted to scan the surface from the same location before to and after plasma treatment. Using the implant jig and the navigation camera installed inside the SEM, we were able to find the location scanned before plasma treatment.

In order to observe the morphology of cells attached to the implant, we first attached cells on the implant samples and fixed them to fluorescently label them. To attach cells on the implant surface, we followed the same protocol utilized in cell proliferation process. After 24 h of incubation in cell culture media with cells adhere to the implants, the implants were rinsed with PBS and immersed in 3.7% formaldehyde solution. After 10 min in the 0.2% triton X-100 solution, the implants were rinsed three times with PBS. Then, the implants were reacted with rhodamine phalloidin (Invitrogen, #R415) and hoechst 33,342 (Invitrogen, #H1399) to label the actin and nucleus of the attached cells, respectively. A widefield fluorescence microscope (Leica Dmi8) was used to image the cells adhered to the implants at magnification of 5x.

## Results and discussion

**Fig. 2:**
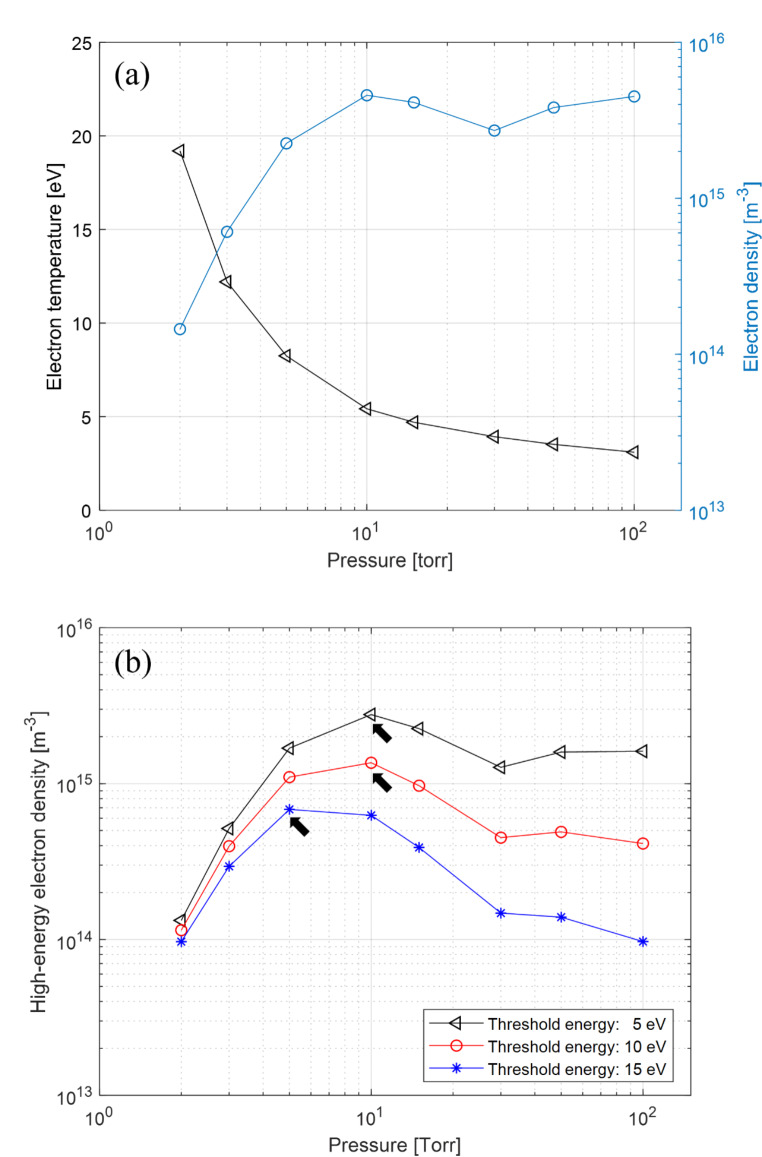
Results of numerical investigation of **a** electron temperature and density, and **b** the effective electron density for different threshold energies of 5 eV (black triangles), 10 eV (red circles), and 15 eV (blue asterisk) as a function of discharge pressure

Through numerical investigation, the spatiotemporal behavior of electrons in plasma was evaluated. More specifically, electron temperature and density were investigated to find the optimal pressure conditions to effectively remove hydrocarbon contaminants from the implant surface. As shown in Fig. 2a, the electron temperature decreases as the pressure increases. Although the electron density increases with increasing pressure, it is saturated at approximately 10 Torr, and it is expected that the optimized plasma conditions are attained at the saturation pressure condition with high electron temperature and density. The threshold energy for hydrocarbon dissociation is approximately 20 eV, and methane is the simplest chemical hydrocarbon, with a high threshold energy of 12.63 eV, and its dissociation reaction in the DBD plasma is as follows:

CH_4_ + e → CH_4_^+^ + 2e (9).

**Fig. 3:**
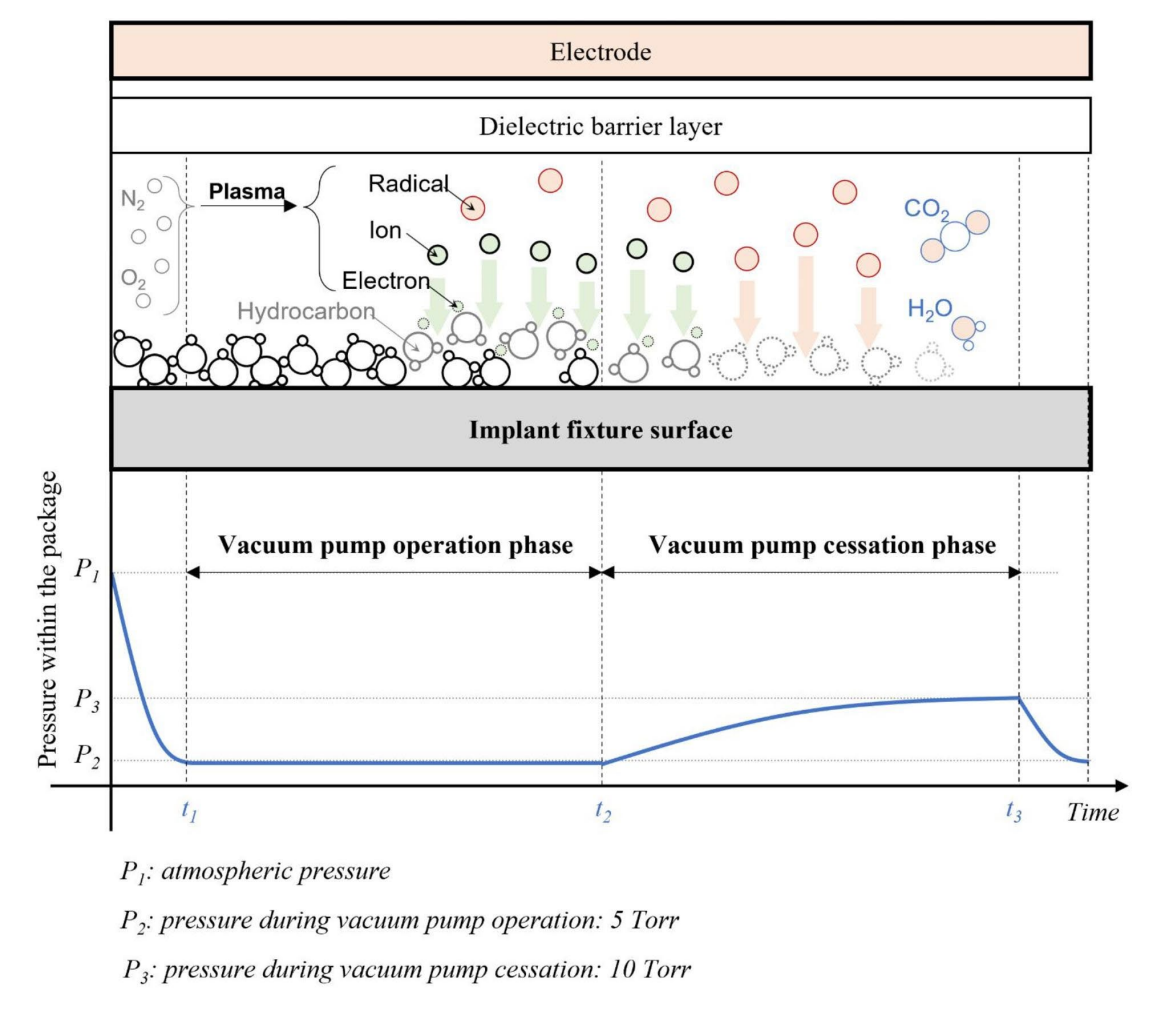
Process configuration for plasma treatment: During the vacuum pump operation phase, plasma is discharged at a pressure as low as 5 torr to weaken the hydrocarbon contaminations that are strongly bound to the implant surface. During the vacuum pump cessation phase, plasma is discharged while the pressure rises to approximately 10 torr to remove surface contaminants by increasing the number of effective electrons

The electrons are considered to have a Boltzmann distribution in this numerical probe. The number of effective electrons can be estimated as the electrons with an energy greater than the threshold energy in the electron energy distribution function (EEDF). The effective number of electrons with various threshold energies of 5, 10, and 15 eV was evaluated as shown in Fig. 2b. As marked with black arrows in Fig. 2b, the optimimal pressure is 10 Torr for dissociating hydrocarbon bondings with lower threshold energies of 5 and 10 eV, whereas a lower pressure of 5 Torr is more appropriate for cracking hydrocarbons with a higher threshold energy of 15 eV. In terms of the bonding dissociation energy, it appears advantageous to discharge plasma at 5 Torr to generate more active electrons with an energy of approximately 15 eV in the plasma. In terms of the total number of effective electrons, however, plasma discharged at 10 Torr seems to contain more effective electrons than 5 Torr (Fig. 2b). Therefore, to take advantage of each pressure condition, we devided the plasma treatment processed into two phases with two different pressure conditions as shown in Fig. 3: (1) vacuum pump operation phase, and (2) vacuum pump cessation phase. Through this two-phase surface treatment strategy, we first tried to weaken the strongly bounded hydrocarbon contaminants and then increased the number of the effective electrons to attempt fast and effective surface treatment.

**Fig. 4:**
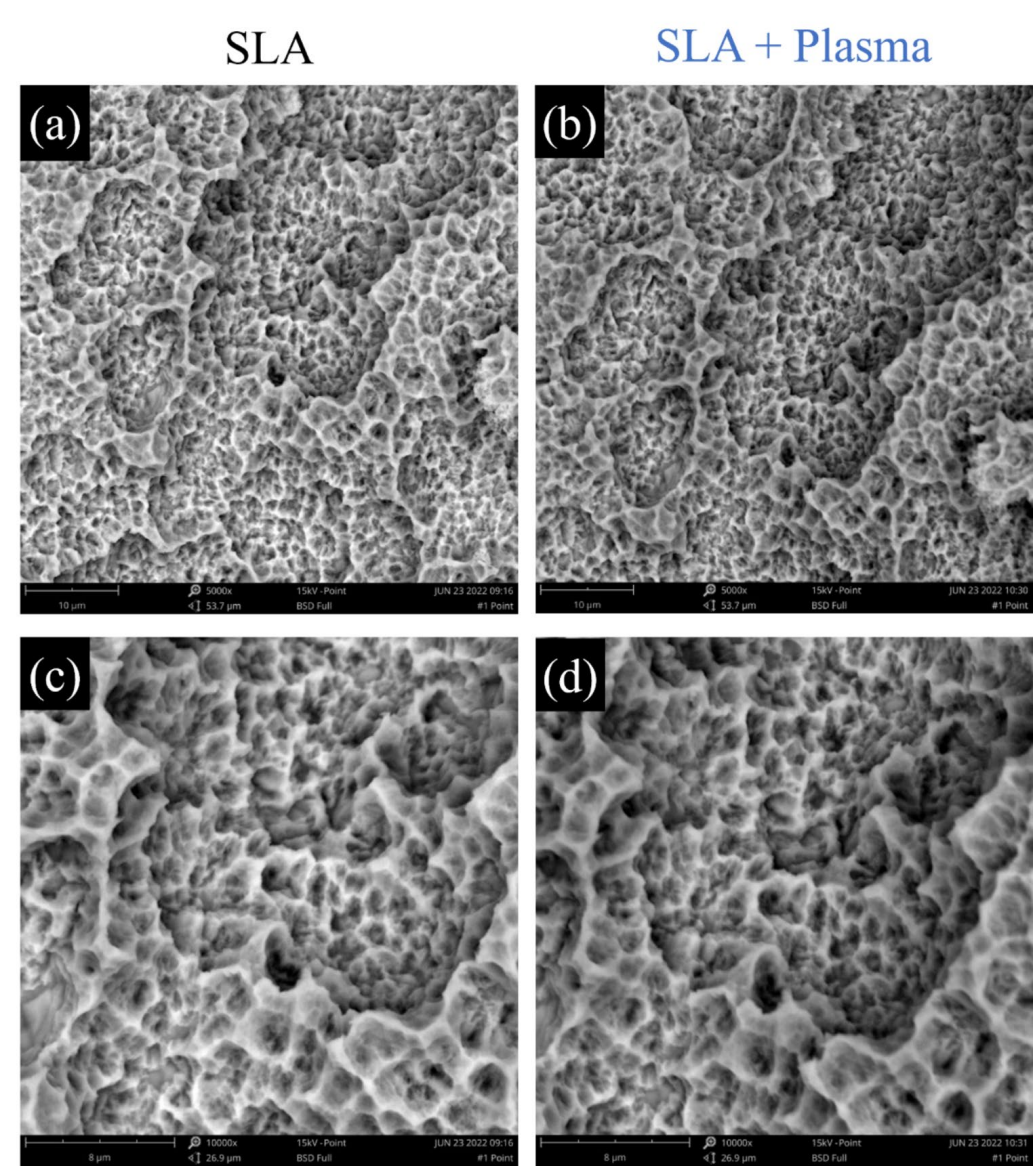
The SEM images of the implant surface **a, c** SLA and **b, d** SLA + Plasma sample. (**a** and **b** are magnified by 5000x, and **c** and **d** by 10,000x)

The surface of SLA and SLA + Plasma was scanned with SEM to confirm wheather plasma treatment causes any physical changes to the implant surface. As can be seen in the the 5,000x and 10,000x images in Fig. 4, there is no noticeable difference between the surface condition before and after plasma treatment. More importantly, no damage such as cracks or corrosion sites was identified on the implant surface after plasma treatment. The macro- and micro-roughness in SLA surface is important for osseointegration, and these results demonstrate that plasma treatment maintain the unique topography of the SLA imaplant surfaces without affecting the intrinsic surface of the implant (Fig. 4).

**Fig. 5:**
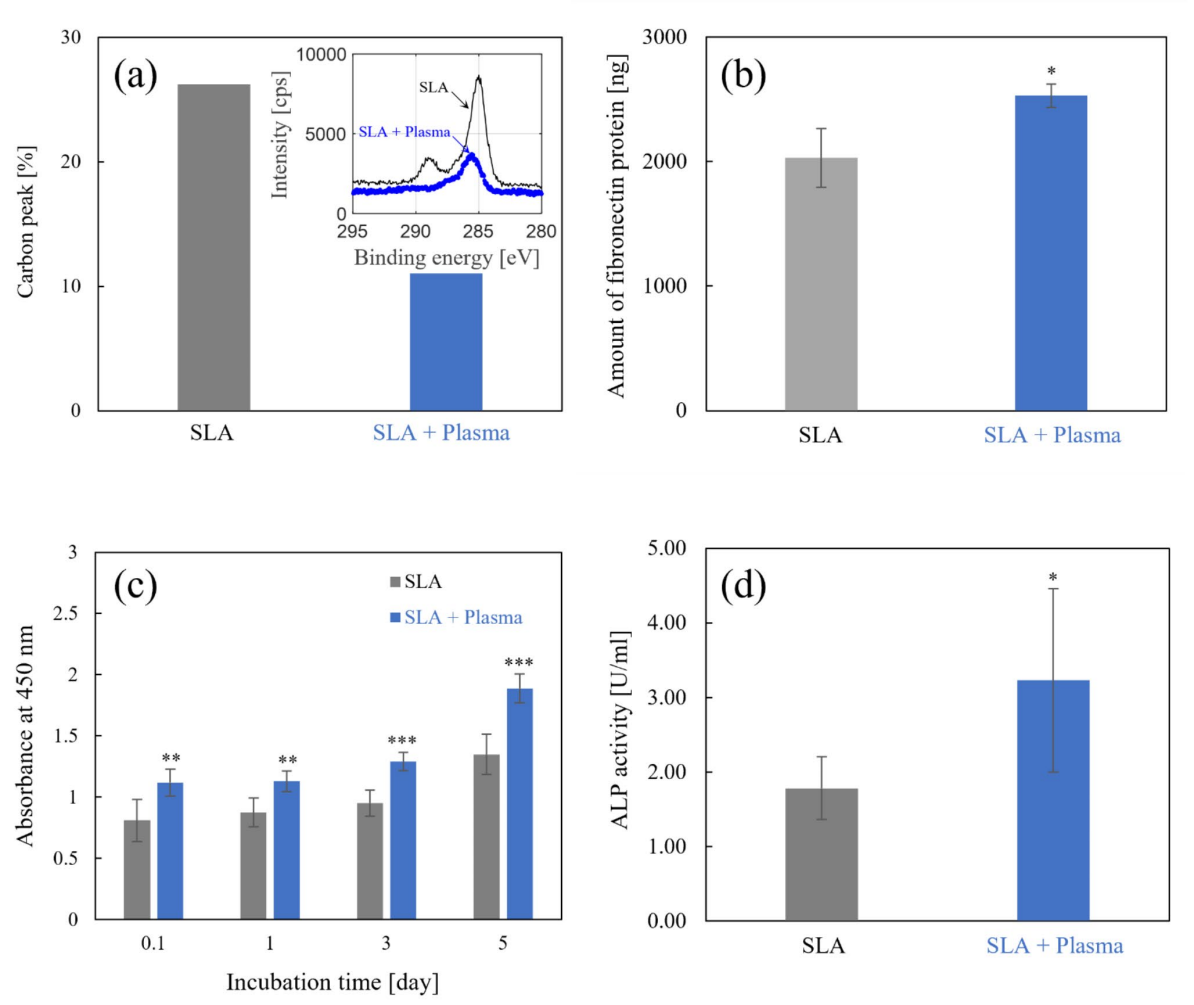
Results of **a** XPS analysis, **b** protein adsorption, **c** cell proliferation, and **d** ALP activity for SLA and SLA + Plasma samples. * P < 0.05, ** P < 0.01, and *** P < 0.001 (Unpaired student’s t-test. Each SLA + Plasma data was compared to the corresponding SLA data.)

The degree of hydrocarbon contamination was determined by X-ray photoelectron spectroscopy, with an energy peak at 285 eV representing the atomic percentage of carbon. It can be seen that the SLA and SLA + Plasma have carbon percentages of 26.2% and 11.0%, respectively, demonstrating that more than 58% of the hydrocarbons on the implant surface are eliminated by the plasma treatment as shown in Fig. 5a. In previous study, it has been reported that protein adsorption to the implant surface increases with a decreasing number of carbon atoms on the surface, indicating a strong negative correlation with a high coefficient of determination (R^2^ = 0.930) between the number of carbon atoms and the amount of adsorbed protein on the implant surface [21]. Similarly, when carbon is gradually eliminated, osteoblast adhesion grows substantially, and the amount of hydrocarbon is also known to be strongly related to cell adhesion rates. Therefore, we use proteins and cells to perform in vitro experiments to identify the effects of plasma-treated surfaces on osseointegration efficiency.

Fibronectin is used in the protein adsorption experiments. When a titanium implant is placed into a bone, protein adsorption occurs on the implant surface as the first physiological phenomenon when it comes into contact with physiological fluids around the site. Among the numerous extracellular matrix (ECM) proteins, fibronectin, in particular, plays an important role in promoting cell adhesion and proliferation by providing an integrin-binding site [31]. We compared the amount of protein adsorbed to SLA and SLA + Plasma surfaces to investigate the effects of plasma treatment on fibronectin adsorption. As shown in Fig. 5b, the amount of proteins adsorbed to the surface of the SLA and SLA + Plasma is measured to be 2,029 ± 236.4 and 2,529 ± 95.7 ng, respectively. Plasma treatment appears to increase protein adsorption to the implant surface by 24.6%.

The number of cells on each implant surface is then measured using a microplate reader at a wavelength of 450 nm. As shown in Fig. 5c, the number of cells attached to the implant surface is approximately 38.5% higher in the SLA + Plasma than in the SLA immediately after the 2-hour time point. This implies that plasma treatment significantly enhances the cell adhesion efficiency. Also, it can be seen that the number of cells in the SLA + Plasma group is approximately 40.2% higher than that in the SLA group after 5 days of incubation, confirming that cells proliferate better on plasma-treated surface (Fig. 5c).

ALP activity is then evaluated after 7 days of culture to assess the level of differentiation. ALP is generally used as an initial marker of osteogenic differentiation, and high ALP activity indicates that cells are more capable of differentiation and functioning as osteoblasts. The ALP activity of the SLA and SLA + Plasma groups is 1.78 ± 0.42 and 3.23 ± 1.23 unit/ml, respectively, as shown in Fig. 5d, demonstrating that ALP activity in the SLA + Plasma group is approximately 81.5% higher than that in the SLA group.

**Fig. 6:**
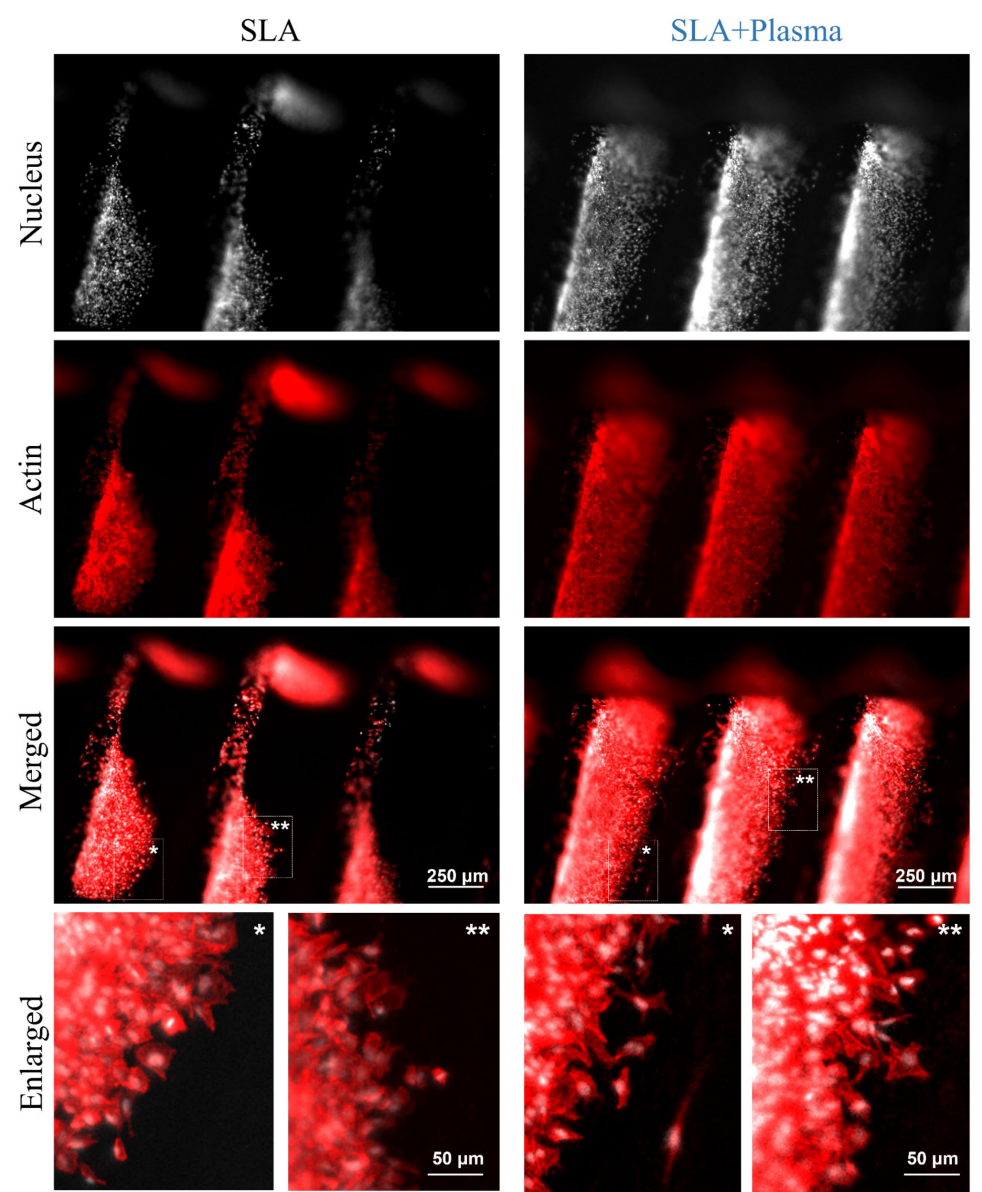
Immunofluorescence images of cells on surface of SLA and SLA + Plasma. Nuclei are shown in gray and actin cytoskeletons in red. Merged represent the overlaid images of actin (red) and nucleus (gray) together. Enlarged represents the enlarged images of the location of * and **

Then, to observe cells on implant surfaces, we attached cells to the implant surfaces, and incubated for 24 h before fixing them. To clearly observe the distribution and morphology of cells, cell nucleus and actin were labeled. In Fig. 6, actin and nucleus are shown in red and gray color, respectively. As can be ceen in Fig. 6, cells tend to attach and proliferate along the thread of the implant in both the SLA and SLA + Plasma samples. However, on the SLA surface, distribution of cells are not uniform and large patches without cells appear in some locations, whereas on the SLA + Plasma surface, cells are uniformly attached to the implant surface and cell density was much higher than on the SLA surface. Furthermore, the enlarged images in Fig. 6 reveal that cells in SLA + Plasma spread somewhat better than cells in SLA, which appear more rounded. This finding reflects the results of the cell proliferation experiment well.

## Conclusion

A plasma treatment source is designed to discharge a cylindrical plasma on the implant surface to reduce hydrocarbon impurities. Based on the numerical investigations, we determined the plasma discharge conditions. Given that there are more high-energy electrons at 5 Torr and more total effective electrons at 10 Torr, a two-step surface treatment strategy has been adopted. By the following in vitro experiments using osteoblast cells, it was confirmed that this two-step plasma treatment on the implant surface efficiently eliminates the hydrocarbon, enhancing protein adsorption and improving cell adhesion, proliferation, and differentiation. Considering the effect of improving osseointegration and the flexibility of equipment design, we expect that this vacuum plasma technology can expand its application to other commercially available implants.

## Data Availability

The data that support the findings of this study are available from the corresponding authors upon reasonable request.
